# Sex differences in the sensitization of prenatally programmed hypertension

**DOI:** 10.3389/fphys.2025.1589615

**Published:** 2025-04-28

**Authors:** Baojian Xue, Alan Kim Johnson, Alexander G. Bassuk

**Affiliations:** ^1^ Stead Family Department of Pediatrics, Roy J. and Lucille A. Carver College of Medicine, University of Iowa, Iowa, IA, United States; ^2^ Department of Psychological and Brain Sciences, College of Liberal Arts and Sciences, University of Iowa, Iowa, IA, United States; ^3^ The Iowa Neuroscience Institute, Roy J. and Lucille A. Carver College of Medicine, University of Iowa, Iowa, IA, United States; ^4^ The Department of Neurology, Roy J. and Lucille A. Carver College of Medicine, University of Iowa, Iowa, IA, United States

**Keywords:** sex differences, sensitization, blood pressure, prenatal programming, renin-angiotensin system, inflammation

## Abstract

Studies have demonstrated that there are sex differences in the timing of onset and severity of prenatally programmed hypertension, with consistently milder phenotypes observed in females relative to male offspring. However, the root cause(s) for these sex-specific effects is unknown. Activation of the renin-angiotensin system (RAS), elevated oxidative stress and inflammation, and sympathetic hyperactivity in the cardiovascular organs and cardiovascular regulatory systems, are all involved in the pathogenesis of hypertension. Sex hormones interact with these prohypertensive systems to modulate blood pressure, and this interaction may lead to a sex-specific development of programmed hypertension. A more complete understanding of the functional capabilities of the sex hormones and their interactions with prohypertensive factors in offspring, from early life to aging, would likely lead to new insights into the basis of sex differences in programmed hypertension. Recently, we have discovered that sex differences also occur in the sensitization of offspring hypertension as programmed by maternal gestational hypertension and that this requires the brain RAS and proinflammatory factors. In this review, we will discuss the possible mechanisms underlying sex differences in sensitization to hypertension in the offspring of mothers exposed to various prenatal insults. These mechanisms operate at various levels from the periphery to the central nervous system (e.g., blood vessel, heart, kidney, and brain). Understanding the sex-specific mechanisms responsible for the sensitized state in offspring can help to develop therapeutic strategies for interrupting the vicious cycle of transgenerational hypertension and for treating hypertension in men and women differentially to maximize efficacy.

## Introduction

The Developmental Origin of Health and Disease (DOHaD) theory (i.e., Barker hypothesis) was first proposed by David Barker, who had discovered the association between lower birth weight and higher mortality from ischemic heart disease (Barker et al., 1989). Subsequently, mounting human epidemiological evidence and animal studies indicated a link between *in utero* adverse stimuli during gestation and an increased risk of hypertension later in life (Arima and Fukuoka, 2020; Falkner et al., 2024; Warrington et al., 2024). The sex differences of prenatally programmed hypertension in offspring have been reported in many different animal models inducing hypertension through a variety of drivers, such as hypertensive disorders during pregnancy, nutritional deficits/excess, uteroplacental perfusion insufficiency, glucocorticoids, nicotine, testosterone treatment or hypoxia during pregnancy (Tomat and Salazar, 2014; Dasinger and Alexander, 2016; Dasinger et al., 2016; Xue et al., 2018; Xue et al., 2021; Xue et al., 2022a; Drury et al., 2024; Darlas et al., 2025). Most of these studies observed that male offspring develop hypertension, whereas female offspring seem to be protected, suggesting a role for the sex hormones such as testosterone and estrogen in modulating the long-term changes in blood pressure (BP) in response to these prenatal insults (Tomat and Salazar, 2014; Dasinger and Alexander, 2016; Drury et al., 2024). The etiology of prenatally programmed hypertension is multifactorial and may include reduced nephron number, endothelial dysfunction, activation of the sympathetic nervous system (SNS) and the renin-angiotensin system (RAS), and elevated oxidative stress and inflammation as well as epigenetic mechanisms (Tomat and Salazar, 2014; Dasinger and Alexander, 2016; Dasinger et al., 2016; Johnson and Xue, 2018; Liu and Liang, 2019; Xue and Johnson, 2023; Drury et al., 2024; Tain and Hsu, 2024). Sex hormones interacting with these prohypertensive systems in male and female offspring from early life to aging may reinforce the formation of the sex differences seen in programmed hypertension (Tomat and Salazar, 2014; Dasinger and Alexander, 2016; Dasinger et al., 2016; Drury et al., 2024).

By using an Induction-Delay-Expression paradigm, we demonstrated that the pretreatment with the non-pressor dose of angiotensin (ANG) II given during Induction (1 week) produced a significantly greater pressor response to the subsequent infusion of slow-pressor doses of ANG II during Expression (2 weeks) when compared to rats pretreated with saline during Induction. This differential pressor response is referred to as hypertensive response sensitization (HTRS) (Xue et al., 2012). Thereafter, we have used a prenatal insult model to investigate the pathogenesis of hypertension in offspring; we demonstrated similar sensitizing effects of maternal gestational hypertension on the development of hypertension in offspring when they are challenged as adults (10 weeks of age) with ANG II or a high fat diet (HFD), as “second hits” (Xue et al., 2017; Xue et al., 2021; Xue et al., 2022a). Sex differences in the effects of prenatal insult to the mother on sensitized hypertension expressed in adult offspring were evident (Xue et al., 2018; Xue et al., 2021; Xue et al., 2022a).

In this review by way of background of our and other studies, we will review sex differences in the sensitizing effects of various prenatal insults to development of hypertension in the offspring. In particular, we will discuss the possible mechanisms of action underlying these sex differences at several levels, ranging from peripheral tissues to the central nervous system (CNS), including blood vessel, kidney, heart, and brain.

## Central nervous system mechanisms

Excessive SNS activation, autonomic dysfunction, activation of the RAS and increased inflammation in the CNS have been associated with development of hypertension (DiBona, 2013; Johnson and Xue, 2018; Xue et al., 2020). Numerous clinical and experimental studies have shown that various prenatal insults can enhance the impact of these central prohypertensive systems. In other words, such prenatal insults can create a sensitized state in the CNS that facilitates the development of prenatally programmed hypertension in offspring later in life (Jones et al., 2007; Tomat and Salazar, 2014; Johnson and Xue, 2018; Zhang et al., 2018; Lim et al., 2021).

Adult obesity is a major risk factor for hypertension, with increased SNS activity serving as the link between the increased adiposity and the elevated BP (Da Silva et al., 2013). For example, leptin is an adipokine that elevates BP via central activation of the SNS, serving as a key mediator for obesity-related hypertension (Da Silva et al., 2013; Taylor et al., 2014). Likewise, it has been established that HFD-induced maternal obesity leads to hypertension in the offspring that is also of sympathetic origin. This was demonstrated by the elevated cardiovascular stress responses to restraint, the enhanced pressor effects of a leptin challenge, the increased sympathetic component of heart rate variability and reduced baroreflex sensitivity in offspring (Prior et al., 2014; Taylor et al., 2014; Gemici et al., 2021).

Perhaps not surprisingly, in animal models, the hypertension in the adult arises from a direct influence of maternal obesity/HFD on the development of BP regulatory pathways in the fetus (Samuelsson et al., 2010). Samuelsson and colleagues support this notion by demonstrating not only that HFD-induced maternal obesity results in a significant increase in BP accompanied by elevated SNS activity in rat offspring as adults, but also that these two effects are already established in the juvenile offspring of obese dams (at 30 days of age). This indicates that the sympathetic overactivation and hypertension arise as a direct consequence of *in utero* exposure to maternal obesity (Samuelsson et al., 2010). Furthermore, there are marked changes in anxiety and spatial learning in offspring from obese dams, and these effects are all observed in adulthood, even after the pups are placed on standard chow at weaning, confirming that these outcomes are programmed early in life (Bilbo and Tsang, 2010). Finally, offspring exposed to maternal obesity/HFD show sex-specific changes in metabolic, behavioral, and BP regulation involving activation of SNS and RAS, hyperleptinemia and increased neuroinflammation that persist into adulthood (Samuelsson et al., 2016; Gemici et al., 2021; do Carmo et al., 2023).

Leptin, acting on melanocortin four receptors (MC4R) in the paraventricular nucleus of hypothalamus (PVN), appears to be required for early-life programming of hypertension arising from either maternal obesity or neonatal hyperleptinemia (Samuelsson et al., 2016). Using transgenic technology to restore MC4R in the PVN of MC4R knockout mice, Samuelsson and colleagues reported that neonatal hyperleptinemia due to maternal obesity induces persistent changes in the central melanocortin system resulting in sympathetic hyperactivity, thereby contributing to offspring hypertension (Samuelsson et al., 2016). Furthermore, do Carmo et al. (2023) found that the male and female offspring of dams with maternal obesity were a greater risk for developing hypertension when the offspring were also kept on the same obesogenic diet, and crucially, found that offspring showed greater reduction in BP during MC4R blockade. However, only the male offspring from obese dams exhibited elevated BP when fed a normal diet and they showed significant BP reduction after adrenergic and ANG II type I receptor (AT1R) blockade. These results confirmed that brain leptin-mediated SNS hyperactivity contributes to the sensitized BP response in obese offspring from obese dams and indicated a sex difference in the mechanisms of BP sensitization in male and female offspring of obese dams (do Carmo et al., 2023).

Obesity is also characterized as a systemic inflammatory condition. Studies showed that microglial activation markers and expression of proinflammatory genes were basally increased in the brain nuclei of neonates from obese dams (Bilbo and Tsang, 2010; Zhang et al., 2018; Wang et al., 2020; Wijenayake et al., 2020). The PVN, amygdala, hippocampus, and prefrontal cortex were among the affected nuclei. Dudele et al. demonstrated that offspring exposed solely to maternal inflammation resemble those born to obese dams. The similarities between offspring subjected to maternal lipopolysaccharide (LPS) or a HFD provide experimental evidence that inflammation is likely to be a key programming factor in pregnancies produced by obesity/HFD (Dudele et al., 2017; Deng et al., 2018). Our studies also confirmed that maternal HFD modulates the brain RAS, oxidative stress, and proinflammatory cytokines (PICs) that alter the actions of ANG II and TNF-α and sensitize the ANG II-elicited hypertensive response in adult offspring. Systemic inhibition of the RAS and PICs can block maternal HFD-induced sensitization of ANG II hypertension, which is associated with attenuation of brain RAS and PIC expression in offspring (Zhang et al., 2018; Wang et al., 2020). Notably, male HFD offspring showed greater proinflammatory gene expression, whereas female HFD offspring exhibited increased anti-inflammatory gene expression in response to simultaneous cortisol and LPS administration. These findings suggest that exposure to maternal HFD leads to sex-specific changes that alter inflammatory responses in the brain (Dudele et al., 2017; Wijenayake et al., 2020). Blood-borne PICs induce a pressor response and sympathetic activation, operating on the brain cardiovascular nuclei to increase RAS activity and inflammation (Wei et al., 2013; Wei et al., 2015; Wei et al., 2018). Estrogen is anti-inflammatory at several levels, including immune cells, adipose tissue, and the brain (Shi et al., 2020). Considering these observations, it is likely that increased PICs in the brains of both male and female offspring induced by prenatal insults including maternal HFD/diabetes may be one of the origins of SNS activation. Since the enhanced brain proinflammatory response and activation of SNS programmed by prenatal insults are sex-specific, they eventually lead to a sex difference in the development of hypertension.

Age may exert a secondary impact on the development of programmed hypertension (Virdis et al., 2011). Intapad et al. showed that growth-restricted male offspring exhibit an increase in BP after puberty, whereas BP is normalized after puberty in growth-restricted female offspring (Alexander, 2003). However, BP was significantly elevated in growth-restricted female offspring by 12 months of age, which was accompanied by increased circulating leptin. This effect could be abolished by bilateral renal denervation. These data indicate that age induces increases in visceral fat and circulating leptin that result in a significant increase in BP in growth-restricted female offspring, with the renal nerves serving as an underlying mechanism (Intapad et al., 2013). Renal denervation also abolishes hypertension in growth-restricted male rats at 3 months of age, further suggesting an important role for activation of the SNS in the etiology of programmed hypertension and it age-dependent and sex-specific manifestations (Alexander et al., 2005). Consistent with the effect of aging on BP in the growth-restricted female offspring, maternal HFD renders the offspring metabolically imbalanced and impairs their ability to cope with a HFD when challenged during aging. The metabolic effects of HFD challenge were more profound in female offspring (Sadagurski et al., 2019) that exhibited more significant changes in blood-brain barrier (BBB) permeability and hypothalamic inflammation compared to male animals (Contu et al., 2019). Moreover, sex differences in hypothalamic estrogen receptor α (ER-α) expression levels were lost in female offspring upon HFD challenge, supporting a link between ER-α levels and hypothalamic inflammation in offspring. These studies highlight the programming potential of hypothalamic inflammatory responses, loss of BBB integrity and maternal obesity, especially in aging females (Contu et al., 2019; Sadagurski et al., 2019).

The RAS is a systemic hormonal regulator of vasoconstriction, aldosterone production, and SNS activity that directly regulates BP. The RAS exhibits autocrine and paracrine capabilities in numerous organs including the brain (Nakagawa et al., 2020). It has been demonstrated that the RAS is involved in prenatal programming of sympathetic overactivity and hypertension. For example, studies show that hypertension induced by maternal protein restriction is associated with an enhanced AT1R expression in several brain nuclei and that the intracerebroventricular administration of an AT1R blocker results in a significant reduction in BP (Pladys et al., 2004; Mizuno et al., 2014). Blockade of the RAS also abolishes hypertension in growth-restricted adult rats exposed to placental insufficiency (Ojeda et al., 2007; Ojeda et al., 2010). In our studies with the ANG II-induced maternal hypertension model, we found that adult male offspring exhibited upregulated expression of both RAS components as well as upregulation of PICs in the lamina terminalis (LT) and PVN. The LT and PVN are forebrain structures with key roles in sodium and water homeostasis and regulation of the cardiovascular system. These adult male offspring also displayed HTRS to a slow-pressor dose of ANG II or to a switch to a HFD post-weaning, when compared with the offspring of normotensive dams (Xue et al., 2017; Xue et al., 2021). A compromised BBB, elevated brain reactivity to pressor stimuli and augmented sympathetic drive to the cardiovascular system likely contributed to the HTRS (Xue et al., 2021). However, this maternal hypertension-induced HTRS was sex-specific, as intact female offspring exhibited an attenuated increase in BP when compared to male offspring (Xue et al., 2018; Xue et al., 2022a). Importantly, different central mechanisms were responsible for the sex differences in the HTRS. Leptin was involved in the expression of HTRS induced by both maternal hypertension and post-weaning HFD feeding in male offspring, but not in females (Xue et al., 2021; Xue et al., 2022a), while antihypertensive components of the RAS, such as angiotensin converting enzyme 2 and AT2R, play a protective role in antagonizing the expression of the HTRS in females (Xue et al., 2014; Xue et al., 2021; Xue and Johnson, 2023).

It is well established that sex hormones such as estrogen and testosterone, are involved in sex differences in the development of hypertension. In our previous study, we demonstrated that relative to males, females were protected against the induction of sensitized hypertension, whereas sensitized hypertensive response was enhanced in male and ovariectomized (OVX) female rats. Central administration of estrogen in either male or OVX female rats during induction blocked ANG II-induced sensitization of hypertension (Xue et al., 2014). Similarly, central Cytochrome P450 1B1 (CYP1B1)-estradiol metabolite, 2-Methoxyestradiol, protects from neuroinflammation and hypertension in female mice (Singh et al., 2020b). In contrast to the protective effects of female sex hormones against the development of hypertension, chronic dihydrotestosterone (DHT) treatment in female rats induced an increase in BP through activation of the SNS and hypothalamic MC4R (Maranon et al., 2015). Testosterone-CYP1B1-generated metabolite 6β-hydroxytestosterone, most likely in the PVN via androgen receptor and G protein-coupled receptor C6A, elicited an increased reactive oxygen species (ROS) production, activation of microglia and astrocyte, and elevated neuroinflammation, contributing to ANG II-induced hypertension in male mice (Singh et al., 2020a). There results indicate that sex hormones and their metabolites act through CNS to contribute to sex differences in the development of hypertension in adult animals. However, few studies have explored pathways and mechanisms through which these sex hormones and their metabolites regulate BP in offspring exposed to various prenatal insults, and further investigations are warranted in the future.

Collectively, the studies highlight the pathogenesis of elevated CNS activity programmed by prenatal insults, which involve the leptin/MC4R pathway, a compromised BBB, central activation of the RAS and increased inflammation, either alone or synergistically. These developmental origins of SNS hyperactivity eventually alter the offspring’s phenotype so as to sensitize their response to prohypertensive agents and/or HFD challenge (i.e., second hits), thereby developing hypertension in adulthood. Sex hormones and aging play an important role in this sensitization process resulting in sex-specific development of programmed hypertension.

## Kidney mechanisms

Epidemiological and animal studies have shown that various prenatal insults all lead to low-birth-weight neonates with increased risk for chronic kidney disease and hypertension (Baum, 2010; Baum, 2018). The kidney dysfunction and elevation of BP that are caused by prenatal insults involve a low nephron endowment, dysregulation of the systemic and intrarenal RAS, increased renal sympathetic nerve activity, and increased tubular sodium transport (Moritz et al., 2009; Lankadeva et al., 2014; Singh and Denton, 2015). Thus, kidney dysfunction plays an important role in generating and maintaining prenatally programmed hypertension in humans and in animal models. Sex difference in the timing of onset and severity of hypertension after prenatal programming also may reflect, in part, sex-specific differences in kidney development and/or the different effects of sex hormones on renal function (Moritz et al., 2010; Dasinger and Alexander, 2016; Drury et al., 2024).

Since nephrogenesis in rodents is not completed until postnatal day 10, the kidney can be programmed not only by various intrauterine perturbations but also by neonatal insults (Baum, 2010; Yim and Yoo, 2015; Baum, 2018). Woods et al. reported that nephron number is reduced in male, but not in female, offspring of low protein dams, and that the formation of a low nephron endowment results in impaired renal function, which in turn contributes to the development of hypertension only in male offspring (Woods et al., 2010). Similarly, both male and female growth-restricted rat offspring have nephron deficits but only the males develop kidney dysfunction and hypertension (Doan et al., 2021). Maternal low-protein diet, neonatal overnutrition and glucocorticoid exposure have similar effects on the reduction of nephron number and cause similar alterations of glomerular morphology and renal cortical oxidative stress in offspring (McMullen and Langley-Evans, 2005; Pedroza et al., 2019; Alhamoud et al., 2021).

do Carmo et al. (2024) examined the impact of maternal obesity on offspring kidney function, morphology, and markers of kidney damage after acute kidney injury (AKI) induced by ischemia-reperfusion at 24–26 weeks of age. They found an increased mortality rate and worse kidney injury scores after AKI in male offspring from obese dams. Female offspring were protected from major kidney injury after AKI. These results indicate that maternal obesity predisposes offspring to kidney dysfunction that sensitizes responses to ischemia-reperfusion injury in a sex-dependent manner (do Carmo et al., 2024). Similarly, hypoxia during late pregnancy disrupted growth of the kidney, particularly the collecting duct network, in male neonates as early as postnatal day 7. By mid-late adulthood (4–12 months of age), these offspring developed early signs of kidney disease, notably a compromised response to water deprivation. Female offspring showed no obvious signs of impaired kidney development and did not develop kidney disease, suggesting an underlying protective mechanism against the hypoxia insult-induced kidney injury in females (Walton et al., 2018).

The RAS is critical for normal renal development (Moritz et al., 2010). In this regard, administration of an angiotensin-converting enzyme inhibitor or an AT1 receptor antagonist during renal development induces significant decreases in body weight and nephron endowment that lead to deterioration of renal function and a significant increase in BP (Saez et al., 2007; Gilbert and Nijland, 2008; Tomat and Salazar, 2014). McMullen and Langley-Evans found that prenatal low-protein and glucocorticoid exposure induce a similar reduction of nephron number. They also found that there are age- and sex-specific differences in the enhancing effects of these two prenatal conditions on postnatal angiotensin receptor expression (AT1R and AT2R) in the kidney, suggesting that upregulation of the RAS plays an important role in the pathogenesis of programmed hypertension through receptor-mediated changes in ANG II activity (McMullen and Langley-Evans, 2005). In a rat model of intrauterine growth restriction (IUGR) induced by placental insufficiency, BP at 4 months of age is increased in male but not female offspring with a normal glomerular filtration rate (GFR). However, in response to a second hit such as acute ANG II, the GFR is reduced in male, but not in female offspring with IUGR (Ojeda et al., 2010; Ojeda et al., 2013; Intapad et al., 2019). Similarly, administration of a postnatal HFD, as a second hit to prenatal dexamethasone, sex-specifically alters protein profiles in offspring kidneys and increases the vulnerability to prenatal-dexamethasone-exposure-induced programmed hypertension, but only in male offspring (Hsu et al., 2018). These data revealed a role for a sensitized renal response to second hits, such as postnatal acute ANG II or HFD, in the pathogenesis of prenatally programmed hypertension in male, but not female, offspring.

Sex hormones such as estrogen and testosterone interact with the RAS to modulate BP (Tomat and Salazar, 2014). In young male and aging female offspring programmed for hypertension by placental insufficiency, the enhanced responsiveness to acute ANG II is testosterone-dependent, and testosterone plays an important role in maintaining the hypertension through enhancement of intrarenal angiotensinogen (Ojeda et al., 2010; Davis et al., 2019a). In contrast, growth-restricted female offspring are normotensive in adulthood. OVX induces a marked increase in BP that is abolished by RAS blockade, and renal hemodynamic responses to acute ANG II are significantly enhanced in growth-restricted female offspring that had undergone OVX, suggesting that sensitivity to acute ANG II is modulated by ovarian hormones in growth-restricted female offspring (Ojeda et al., 2011; Davis et al., 2019b). In our ANG II-induced maternal hypertensive rat model, we also found sex differences in the maternal hypertension-induced sensitization of ANG II hypertension in offspring. Castration did not alter the hypertensive response to ANG II in male offspring, whereas OVX induced a greater increase in the pressor response to ANG II in female offspring of hypertensive dams compared with female offspring of normotensive dams. Furthermore, either RAS blockade or renal denervation abolished the maternal hypertension-induced sensitization of hypertension in offspring (Xue et al., 2017; Xue et al., 2018). It has been shown that estrogens promote the anti-hypertensive effects of the RAS by enhancing the Ang-(1–7)/AT2R pathway while diminishing the ACE/AT1R pathway (Colafella and Denton, 2018). Therefore, modulation of the renal RAS by estrogen or testosterone serves as a potential mechanism in mediating the sex-specific differences in hypertension in offspring programmed by prenatal insults.

In summary, various prenatal insults lead to a reduced number of nephrons, impaired renal development and function, and disturbance of the RAS activity in the kidney, which may fundamentally lead to a sensitized response to second hits (e.g., ANG II or HFD) that participate in both the development and the maintenance of prenatally programmed hypertension in offspring. Sex hormone interactions with the renal RAS play important roles in the sex-specific processes during the development of programmed hypertension.

## Cardiovascular mechanisms

Alterations in the structure and function of vascular smooth muscle, endothelium, and the heart are involved in the development of prenatally programmed hypertension and of myocardial injury, in which nitric oxide (NO), ROS, RAS activity and β-adrenergic receptors play a mediating role (Tomat and Salazar, 2014). Sex differences in vascular and heart dysfunction in different animal models of prenatal programming are also observed, and sex hormones play a modulatory role in cardiovascular responses to an adverse fetal environment (Chen et al., 2019; Drury et al., 2024).

After maternal malnutrition or fetal glucocorticoid exposure, it is only the male offspring who suffer an increase in BP. Vascular NO and ROS contribute to this sex-specific programmed hypertension. While maternal malnutrition increased both superoxide-mediated vasoconstriction and NO mediated vasodilation, the balance of these factors favored the development of hypertension in males and hypotension in females (Roghair et al., 2009). Moreover, females counteract the adverse effects of maternal malnutrition through the development of a better antioxidant status during the critical developmental window of prenatal life. This early female advantage, together with the ability of estrogen to scavenge free radicals during pregnancy (Reyes et al., 2006), contribute to the milder consequences of programmed hypertension in females (Rodriguez-Rodriguez et al., 2015).

Cardiovascular changes are also involved in other forms of prenatally programmed hypertension. Prenatal nicotine or hypoxia had no effect on baseline BP but caused a heightened vascular response to ANG II and increased the BP in adult male, but not in female, rat offspring. This increase in BP was associated with increased arterial media thickness and the ratio of AT1R/AT2R in the aorta, but not with endothelial NO synthase activity in males. These results suggest that prenatal nicotine or hypoxia exposure alters vascular function via changes in ANG II receptor-mediated signaling pathways in a sex-specific manner (Xiao et al., 2008; Xiao et al., 2014). Estrogen counteracts heightened ROS production, leading to protection of females from prenatal programming of a hypertensive phenotype in adulthood (Xiao et al., 2013; Xiao et al., 2014). In mouse offspring of dams with maternal hypertension induced by vasopressin infusion throughout gestation, a sensitized vascular response to a low dose of ANG II was also evident. Moreover, genetic interference with peroxisome proliferator-activated receptor-γ (PPARγ) specifically in the vascular endothelium of these offspring augmented ANG II-induced endothelial dysfunction. This impairment in endothelial function was attenuated by scavengers of ROS, an effect more prominent in male offspring than female offspring (Nair et al., 2019). In addition, the finding that prenatal nicotine or hypoxia had no significant effect on BP under resting conditions in adult offspring, but enhanced the BP response to ANG II treatment in adult male offspring are consistent with findings in several different animal models including our maternal hypertension model (Peyronnet et al., 2002; Xiao et al., 2009; Intapad et al., 2015; Xue et al., 2018). These data suggest a sensitizing effect of prenatal insults on vascular responses, thereby resulting in the development of hypertension later in life when the subjects encounter second hits.

Similarly, a maternal HFD causes a sex-specific regulation of vascular AT1R and AT2R gene expression through epigenetic DNA methylation, which leads to heightened vascular contraction in adult male, but not female, offspring (Chen et al., 2021). Estrogen plays a key role in this sex difference, as it normalizes vascular dysfunction induced by maternal HFD in female offspring by regulating ATRs, thereby leading to a reduced development of a hypertensive phenotype in adulthood (Chen et al., 2022).

In the rat model using prenatal dietary protein restriction, BP is significantly higher in male than in female offspring. However, OVX induces a significant increase in BP in such females, while estrogen replacement partially reduces the increased BP (Sathishkumar et al., 2009, 2012). It has been shown that prenatal protein restriction leads to compromised ovarian function, thereby contributing to reduced levels of vascular ERα receptors and plasma estrogen, and increased testosterone in female offspring (Leonhardt et al., 2003; Guzman et al., 2006). Therefore, it is likely that prenatal insults could have adversely impacted developing organs (e.g., ovarian dysfunction, and reduced ERα in blood vessels) such that they became less responsive to estrogen. As a result, estrogen replacement reversed only the OVX-induced increase in BP but not that induced by maternal protein restriction (i.e., BP was not recovered to control level) (Sathishkumar et al., 2012). Similar to the aforementioned studies, in our maternal hypertension rat model, for female offspring challenged with postnatal ANG II, OVX increased BP and estrogen replacement partially rescued this effect (Xue et al., 2018).

The sympathetic branch of the autonomic nervous system exerts its predominant impact on cardiomyocytes via β-adrenergic receptors, which can be affected by prenatal programming (Giussani et al., 2012; Harvey et al., 2015). The inotropic and chronotropic responses to the β-adrenergic receptor agonist, isoproterenol, following prenatal protein restriction, were unaltered in female offspring, but significantly higher in male offspring. Estrogen plays a key role in regulation of the expression of β-adrenergic receptors in the heart. Expression of β-adrenergic receptors is upregulated in the OVX rat heart but quickly reversed following estrogen replacement (Elmes et al., 2009). Moreover, male offspring exposed to prenatal hypoxia had an increased susceptibility to ischemic myocardial injury (similar to heart failure involving diastolic dysfunction in adult life) compared with both offspring from healthy pregnancies and with their female counterparts (Shah et al., 2017). These results suggest that prenatal insults such as prenatal low protein or hypoxia, sex-specifically program the sensitivity of β-adrenergic receptors to stimulation and alter AT1R/AT2R expression patterns in the offspring’s heart, which may explain the higher sympathovagal balance and increased susceptibility to ischemia-reperfusion injury in male offspring subject to prenatal insult when compared to female offspring (Elmes et al., 2009; Giussani et al., 2012; Harvey et al., 2015; Xue et al., 2015).

In summary, the prenatal insults induce an increased ratio of AT1R/AT2R, elevated ROS production, and decreased NO bioavailability in conduit and resistance arteries, and these changes act collectively to elicit a sensitized vascular responsiveness, thereby resulting in elevation of the BP. The prenatal insults also program the expression and sensitivity of β-adrenergic receptor in cardiomyocytes to increase susceptibility to ischemic myocardial injury. Female sex hormones, especially estrogen, play a protective role in the prenatal insult-induced sensitization processes, which manifest as cardiovascular dysfunction in a sex-specific manner.

## Alterations and effects of maternal sex hormones in prenatal insults

Sex hormones, including estrogen, progesterone, and testosterone, are essential for the physiological regulation of pregnancy such as vascular adaptations, pregnancy maintenance and labor process. However, low levels of estrogen or high levels of testosterone during pregnancy have been shown to initiate various prenatal insults, such as preeclampsia (Shu et al., 2021; Darlas et al., 2025).

Several clinical studies have documented a significant decrease in the levels of estradiol during preeclampsia (Berkane et al., 2017; Cantonwine et al., 2019). The decrease in estrogen levels is due to alterations in enzyme activities including a decrease in Hydroxysteroid (17-β) dehydrogenase 1 (17β-HSD1, an enzyme converting estrone to estradiol), aromatase (an enzyme converting androgens to estrogens), catechol-O-methyltransferase (COMT, an enzyme for the synthesis of 2-methoxyestradiol) (Taravati et al., 2017; Berkane et al., 2018). The exogenous administration of estrogen normalized the BP and other associated symptoms of preeclampsia in both animal models and preeclampsia patients (Djordjevic et al., 2010; Babic et al., 2018; Lin et al., 2020). These beneficial effects of estrogen involve the activation of GPR30, endothelial NOS and PI3K-Akt signaling pathway, reduced release of inflammatory cytokines and oxidative stress, leading to improved placental perfusion (Shu et al., 2021).

A meta-analysis reveals that expectant mothers with hyperandrogenic polycystic ovary syndrome (PCOS) had increased odds ratios for gestational diabetes mellitus and preeclampsia compared to those with a non-hyperandrogenic PCOS (Guo et al., 2024). Indeed, maternal androgen levels are already elevated in the early second trimester among women who eventually develop preeclampsia. Thus, hyperandrogenism is involved in the pathogenesis of preeclampsia, and may be considered an early risk marker of preeclampsia (Carlsen et al., 2005). Chinnathambi et al. confirmed that increased maternal testosterone in female rats, at concentrations relevant to abnormal clinical conditions, induces blunting of NO-mediated vasodilation and increased vascular resistance, leading to maternal gestational hypertension (Chinnathambi et al., 2013a).

Conditions of excess androgen in women, such as PCOS, often exhibit intergenerational transmission. In a human study, women born to mothers with the highest levels of maternal bioactive androgens demonstrated a 4.84-fold increased odds for having hypertension and were associated with an increased risk for incident metabolic syndrome (Huang et al., 2018). In animal studies, Sherman et al. demonstrated that prenatal exposure to excess androgen negatively impacted cardiovascular function by increasing BP and decreasing heart rate in adult female offspring. Prenatal androgen was also associated with gut microbial dysbiosis. These results suggest that prenatal exposure to hyperandrogenemia in daughters of women with PCOS may lead to long-term alterations in gut microbiota and cardiometabolic function (Sherman et al., 2018). Similarly, male offspring of hyperandrogenemic dams had a normal baseline BP, but an exaggerated pressor response to ANG II infusion, suggesting that adult sons of PCOS mothers may also be at increased risk of cardiometabolic disease (Zuchowski et al., 2021). Further, prenatal testosterone leads to an increase in BP in both male and female offspring but involves a sex-specific mechanism responsible for blunting of endothelial cell-associated relaxation: endothelium-derived hyperpolarizing factor (EDHF)-related in males and NO-related in females (Chinnathambi et al., 2013b). The ACE inhibitor enalapril has a positive influence on endothelial function with improvement in EDHF relaxation (More et al., 2015).

In summary, both decreased estrogen and increased testosterone during pregnancy contribute to the initiation of prenatal insults, particularly preeclampsia, which is associated with detrimental consequences for both mothers and offspring, exhibiting sex-specific cardiovascular dysfunctions.

## Conclusion

**FIGURE 1:**
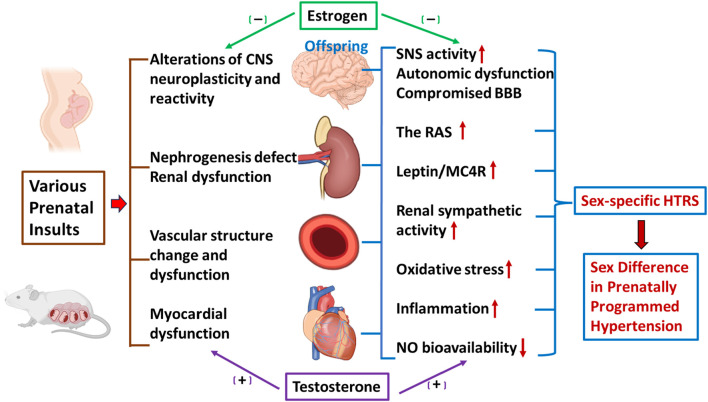
Schematic representation of sex differences in prenatal insult-elicited hypertensive response sensitization (HTRS) and associated peripheral and central mechanisms. The figure shows prenatal insults elicit a sex-specific HTRS (i.e., prenatally programmed hypertension) through cardiovascular dysfunction, nephrogenesis defects, and alterations of central nervous system (CNS) neuroplasticity and reactivity. Decreased nitric oxide (NO) bioavailability, increased oxidative stress and inflammation, activation of the renin-angiotensin system (RAS), disruption of the blood-brain barrier (BBB), and elevation of leptin/melanocortin 4 receptors (MC4R) and sympathetic nervous system (SNS) activity in the peripheral and CNS networks controlling blood pressure (BP) mediate these sensitization processes, in which sex hormones such as estrogen and testosterone, are involved.

In this review, we have described the sex differences in the pathogenesis of prenatally programmed hypertension and the associated peripheral and central mechanisms. The prenatal insults program the cardiovascular system and cardiovascular-regulating organs, such as the blood vessels, heart, kidney, and the CNS, to adversely alter their structures and functions in a way that increases the risk for hypertension in adults. These prenatal and/or early life alterations include reduced nephron endowment, changes in factors that affect endothelial and arterial compliance, and alterations in RAS and SNS activity in the periphery and the CNS. These changes increased the sensitivity of the offspring of mothers exposed to various prenatal insults to a second hit (e.g., stress, HFD or prohypertensive agents), increasing the likelihood that the offspring will develop hypertension later in life. Sex hormone interactions with the prohypertensive systems in the cardiovascular organs play a pivotal role in the pathogenesis of sex-specific and age-dependent hypertension programmed by various prenatal insults (Figure 1). However, despite intense investigation into the mechanisms underlying fetal programming of adult hypertension, there is still no consensus on how these different mechanisms and pathways are programmed *in utero* and how they interact with sex hormones to ultimately lead to a sex-specific increase in BP later in life. Future studies are warranted to investigate the mechanisms involved in initiating programming events *in utero*, and the subsequent sensitization to postnatal challenges (i.e., stresses). Understanding these mechanisms is important for the development of novel, sex-specific strategies for prevention and treatment of hypertension in men and women.

Since the current evidence shows that fetal and early postnatal life are critical developmental windows, one opportunity for interventions to ameliorate prenatally programmed hypertension is around these developmental periods. We and others have demonstrated the beneficial effects of anti-RAS or anti-inflammatory agents administered to mothers or offspring on improving fetal programming outcomes (Mizuno et al., 2014; Xue et al., 2017; Deng et al., 2018; Li et al., 2020). However, these therapies cannot be used during pregnancy or postnatally in human because these treatments are likely to interfere with renal and CNS development in the offspring. Thus, there is great need for developing therapeutic strategies for women exposed to prenatal insults which do not involve the risk of teratological toxicity. Exercise in the mother during pregnancy and in the offspring may be a promising strategy to prevent or reprogram latent HTRS (Kusuyama et al., 2020). Our recent studies showed that voluntary exercise in offspring plays a beneficial role in preventing maternal hypertension-induced HTRS elicited by postweaning HFD (Xue et al., 2022b). Further studies are needed to investigate the underlying mechanisms involved in the positive reprogramming effects of “good” therapeutic strategies, not limited to exercise, that will improve maternal and offspring health and prevent the vicious cycle of transgenerational hypertension.
